# Physiological Ripples Associated With Sleep Spindles Can Be Identified in Patients With Refractory Epilepsy Beyond Mesio-Temporal Structures

**DOI:** 10.3389/fneur.2021.612293

**Published:** 2021-02-10

**Authors:** Jonas C. Bruder, Christoph Schmelzeisen, Daniel Lachner-Piza, Peter Reinacher, Andreas Schulze-Bonhage, Julia Jacobs

**Affiliations:** ^1^Department of Neuropediatrics and Muscular Disease, University Medical Center, Freiburg, Germany; ^2^Stereotactic & Functional Neurosurgery, University Medical Center, Freiburg, Germany; ^3^Epilepsy Center, University Medical Center, Freiburg, Germany

**Keywords:** high frequency oscillations, ripples, sleep spindles, epileptic spikes, post-surgical outcome, refractory epilepsy

## Abstract

**Introduction:** High frequency oscillations (HFO) are promising biomarkers of epileptic tissue. While group analysis suggested a correlation between surgical removal of HFO generating tissue and seizure free outcome, HFO could not predict seizure outcome on an individual patient level. One possible explanation is the lack of differentiation between physiological and epileptic HFO. In the mesio-temporal lobe, a proportion of physiological ripples can be identified by their association with scalp sleep spindles. Spike associated ripples in contrast can be considered epileptic. This study investigated whether categorizing ripples by the co-occurrence with sleep spindles or spikes improves outcome prediction after surgery. Additionally, it aimed to investigate whether spindle-ripple association is limited to the mesio-temporal lobe structures or visible across the whole brain.

**Methods:** We retrospectively analyzed EEG of 31 patients with chronic intracranial EEG. Sleep spindles in scalp EEG and ripples and epileptic spikes in iEEG were automatically detected. Three ripple subtypes were obtained: SpindleR, Non-SpindleR, and SpikeR. Rate ratios between removed and non-removed brain areas were calculated. We compared the distinct ripple subtypes and their rates in different brain regions, inside and outside seizure onset areas and between patients with good and poor seizure outcome.

**Results:** SpindleR were found across all brain regions. SpikeR had significantly higher rates in the SOZ than in Non-SOZ channels. A significant positive correlation between removal of ripple-events and good outcome was found for the mixed ripple group (r_s_ = 0.43, *p* = 0.017) and for ripples not associated with spindles (r_s_=0.40, *p* = 0.044). Also, a significantly high proportion of spikes associated with ripples were removed in seizure free patients (*p* = 0.036).

**Discussion:** SpindleR are found in mesio-temporal and neocortical structures, indicating that ripple-spindle-coupling might have functional importance beyond mesio-temporal structures. Overall, the proportion of SpindleR was low and separating spindle and spike associated ripples did not improve outcome prediction in our patient group. SpindleR analysis therefore can be a tool to identify physiological events but needs to be used in combination with other methods to have clinical relevance.

## Introduction

Around 30% of patients continue to suffer from epileptic seizures after optimized medical treatment (1). Their best chance to achieve seizure freedom is epilepsy surgery offering success rates of up to 80% (2). Epilepsy surgery aims to resect all epileptic tissue including the seizure onset zone (SOZ), which is defined as the area of the cortex that generates seizures at a given point in time (3). In patients in whom non-invasive diagnostics cannot securely identify epileptic regions, intracranial video-EEG (iEEG) monitoring is considered the gold standard to localize the primary epileptic focus (4).

High frequency oscillations (HFO, ripples: 80–250 Hz, fast ripples: 250–500 Hz) are promising EEG markers of epileptic tissue (5–9). HFO rates were repeatedly shown to be higher in the SOZ (5, 6, 10, 11) and the resection of HFO-generating areas correlated with a good postsurgical outcome in several studies (9, 12, 13). These findings were confirmed by a meta-analysis of Höller et al. reviewing 11 HFO studies (14). Furthermore, HFO were considered superior to spikes in delineating the SOZ by some studies (6, 15). Nevertheless, the question whether epileptic spikes or HFO are more reliable biomarkers of epileptic tissue is still controversial. For instance, Roehri et al. found no benefits in using HFO instead of spikes for delineating the SOZ, especially on a single patient level. Furthermore, the analysis of HFO co-occurring with spikes could improve the delineation of epileptogenic areas (16–18).

Several retrospective analysis have shown that removing HFO-generating areas correlates well with favorable postsurgical outcome in group analyses (19). In the clinical context outcome prediction is only relevant if it can be performed prospectively and on a single-patient basis. Results show that HFO can correctly predict outcome in some but not all patients (12, 13, 20).

Several pitfalls have been identified when using HFO to delineate epileptic areas. One of the most commonly named challenges is the co-existence of physiological and epileptic HFO. As Engel and co-workers pointed out early on, a simple frequency analysis does not allow us to safely separate physiological HFO. Identification of physiological HFO in the human brain is complicated for two reasons. First of all, for ethical reasons all patients investigated with iEEG are suffering from epilepsy and might have widespread brain abnormalities. Identifying clearly healthy brain regions and certain physiological HFO is challenging but can be accomplished as has been recently demonstrated by Frauscher et al. (19). Their atlas of physiological HFO activity suggests that physiological HFOs are visible over most brain regions in agreement with other recent studies that could show physiological HFO activity originating not only from mesio-temporal regions but also from central and occipital regions (21, 22). Identifying HFO in clearly healthy brain tissue however does not help to overcome the second challenge, which is to separate physiological and epileptic HFO in regions of the SOZ and those with clear epileptic activity. In these regions either advanced analysis of HFO frequency and amplitude characteristics (23–25) or coupling analysis to co-occurring EEG phenomena has been successfully used (26, 27).

**Figure 1:**
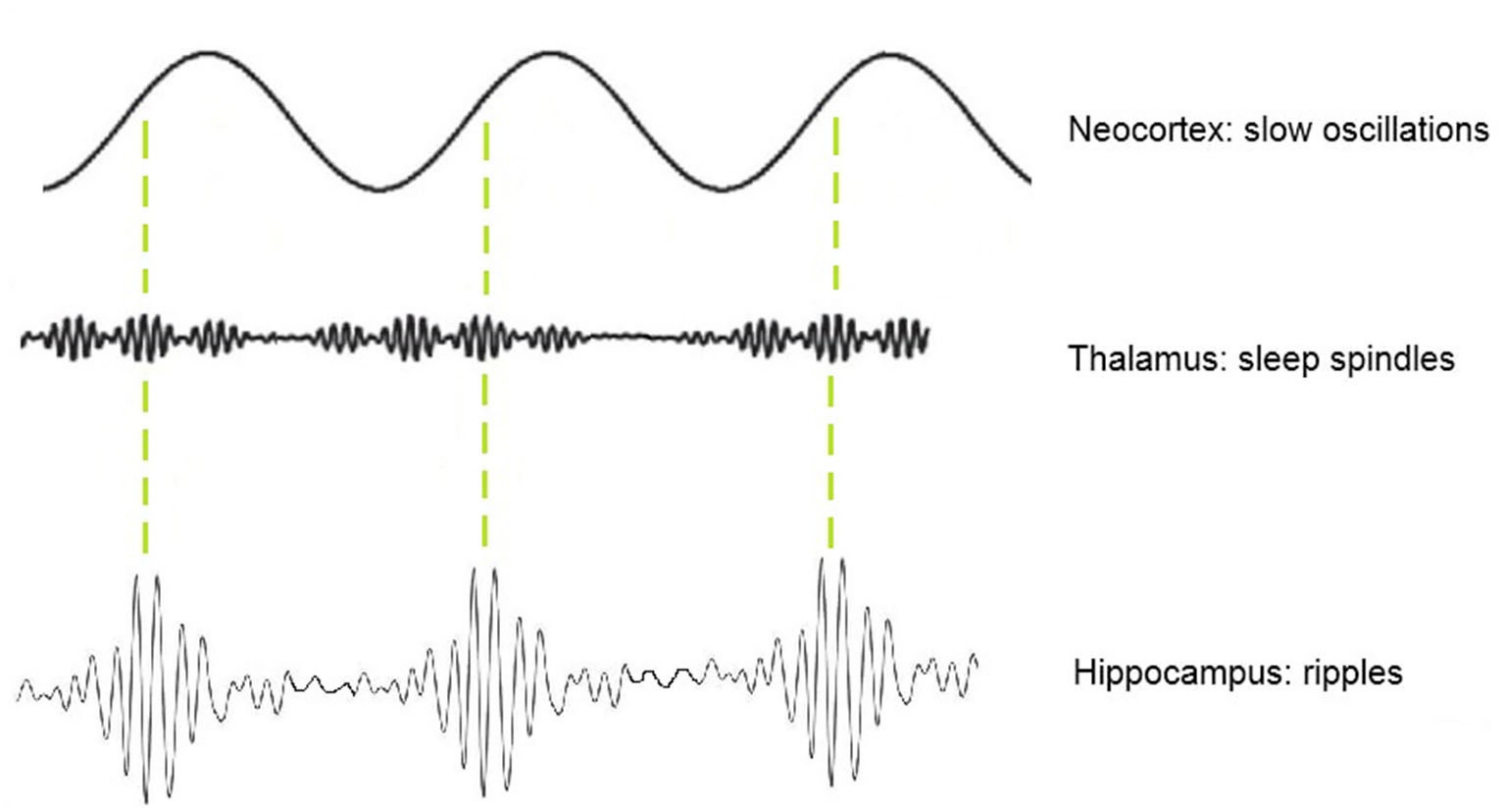
Schematic illustration of thalamic sleep-spindles and hippocampal ripples nesting in the depolarizing up-states of neocortical slow oscillations.

One approach for identifying physiological HFO that has been previously explored by our group is the analysis of spindle–ripple coupling (25). At this point, spindle-ripple association has been shown for physiological ripples in mesio-temporal structures (27–29). Clemens and co-workers stated that thalamo-cortical sleep spindles—functionally linked to periods of reduced sensory input—enable a secure timeframe for information transfer from the HC to the neocortex (30, 31). Ripples nested into single troughs of spindles are believed to enable a temporally synchronized memory-transfer from the HC to neocortical areas for long-term storage (30, 32, 33). The formation of spindle–ripple events is thought to be supported by neocortical slow oscillations (<1 Hz) which organize the occurrence of both thalamocortical spindles and hippocampal ripples (as illustrated in Figure 1). By analyzing the oscillatory features of a mixed group of ripple-range HFO, our group found that HFO associated with sleep spindles have different amplitude features than those with spikes and in the SOZ. Their lower amplitude could be used to separate mesio-temporal ripples from other ripples (25, 34).

It remains unclear, if spindle associated ripples also occur outside mesio-temporal structures and might support other cognitive functions. In the current paper we therefore aim to investigate ripple-spindle association across the brain including temporal neocortical, frontal, parietal, and occipital areas. We hypothesize that sleep spindle-ripple-links might support information transfer across brain regions for different functional purposes. Moreover, we investigate whether systematic separation of ripples associated with spindles and spikes can improve surgical seizure outcome prediction in our patient population.

## Methods

### Patient Selection

One hundred and eight patients received chronic intracranial EEG (iEEG) at Freiburg Epilepsy Center between January 2012 and December 2017. The decision for implantation and the exact placement of the invasive electrodes was solely based on the clinical needs and results of a multidisciplinary surgical case conference. All EEG recordings were evaluated independently of this study by experienced neurophysiologists, who also determined the extent of the SOZ (3). HFO were not clinically used for delineating the epileptogenic area. The study was validated by the Ethics Committee of the Freiburg University Medical Center.

For this study, inclusion criteria were: at least one electrode in the mesio-temporal structures, simultaneous scalp EEG for sleep spindle detection and an EEG sampling rate of 2 kHz. For outcome prediction we also only included patients which underwent surgery after iEEG recording.

### Recording Methods

Intracranial depth electrodes with five to 18 contacts and a diameter of 0.8 mm made of Platinum/Iridium (Dixi Medical, Besancon, France) were implanted. Intracranial EEG was recorded with a digital video system called “Profusion EEG Software” (Compumedics Limited, Abbotsford Victoria, Australia) and sampled with a 2 kHz rate using a digital low-pass filtering with a cutoff frequency of 800 Hz. Ten- to twenty-system scalp EEG combined with electrooculogram and electromyogram was installed the second day after iEEG implantation. The different sleep stages were determined independently from this study by experienced EEG technologists according to the American association of sleep medicine (AASM) guidelines (35).

### EEG Segment Selection

As spikes and HFO occur more frequently in slow-wave-sleep (36) and sleep spindles are found predominantly in slow wave sleep stage N2 (37), we chose N2-EEG periods for all analyses. For each patient 30 min of EEG with at least 60 min distance to epileptic seizures were selected.

The EEG data was transformed into a binary format and high-pass-filtered using the “ASA” (ANT Neuro, Enschede, Netherlands) software via 2nd Butterworth filter with a cut-off-frequency of 0.5 Hz. All files were then converted into “edf”-format for automatic detection.

### Detection and Division of Ripple Subtypes

Automatic detection of ripples and spikes was performed on iEEG, while frontal and parietal sleep spindles were detected on the simultaneous scalp EEG. For both analysis previously published detectors were used (38, 39). These detectors are based on the multivariate classification of iEEG epochs using kernelized support-vector-machines. The features used for the multivariate classification described the amplitude, waveform and frequency characteristics of the iEEG epochs and were also based on the raw, filtered and wavelet-transformed signals. The description of the feature calculation and selection is described in the corresponding publications, as well as the procedures followed for the training, validation and testing of the detectors. We used a custom MATLAB 2018b script to determine ripples coinciding with spikes and sleep-spindles.

The first 5 min of each EEG segment were then visually examined to exclude any EEG artifacts i.e., background noise. Ripples were categorized into four subtypes: all ripples, ripples coincident with scalp sleep spindles (SpindleR), ripples not coincident with scalp spindles (Non-SpindleR) and ripples coincident with epileptic spikes in the same iEEG channels (SpikeR). Ripples coincident with both spindles and spikes were excluded as we were not able to categorize them as either epileptic or physiological.

### Clinical Data

Clinical information on lesion, epilepsy type, EEG, imaging results and postsurgical outcome were collected from the electronic patient record system. All patients had at least 12 months of postsurgical seizure follow-up.

All patients received MRI while the electrodes were in place as well as 3 months after epilepsy surgery. MRI with electrodes in place were used to locate channels and assign them to one brain region. Both MRI were co-registered using SPM software to visualize which contacts were located in the surgical cavity. This analysis allowed us to clearly decide whether a contact was located within or outside the surgical area. In <5% of the channels a clear allocation was not possible and these were excluded from analysis. Examples would be contacts directly located at the border of a resection or in brain areas that can be considered as functionally disconnected after the resection. All EEG-contacts were divided into surgically removed channels (RemCh) or channels remaining after surgical intervention (Non-RemCh).

### Statistical Analyses

**Figure 2:**
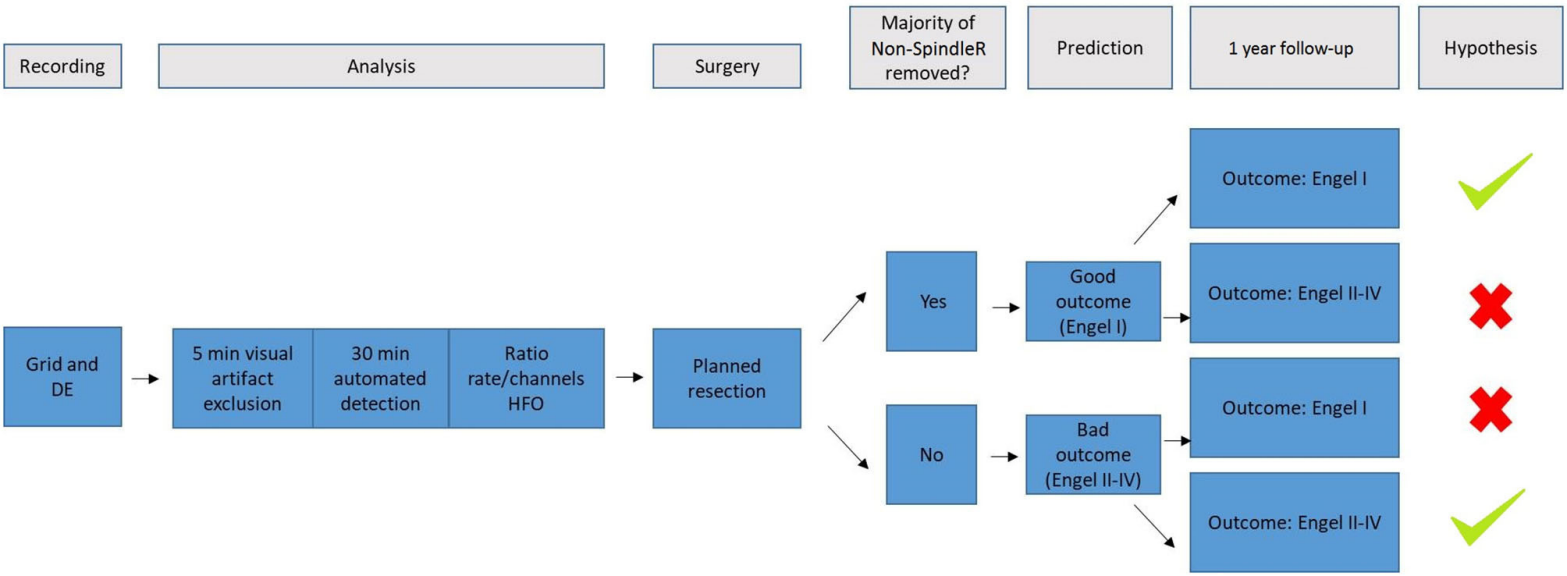
It summarizes the methodological approach of this study. We used scalp and intracranial EEG (DE, depth electrodes) of the video-EEG-monitoring of patients with refractory epilepsy. The raw EEG data was then visually examined to exclude channels in white matter or with too many artefacts. Afterwards, sleep spindles were automatically detected in frontal/parietal scalp EEG contacts; ripples and spikes were automatically detected in intracranial EEG. Rate ratios (mirroring the extent of removal of HFO generating tissue) were calculated for All Ripples, ripples outside spindles (Non-SpindleR) and Spike-Ripples. All included patients received surgery and a 1 year-follow-up. We hypothesized, that the removal of brain tissue generating ripples outside spindles would lead to a good postsurgical outcome after 12 months, whereas the remaining of respective tissue would lead to a bad postsurgical outcome.

Figure 2 summarizes the methodological approach of this study.

### Descriptive Statistics

In our descriptive analysis we examined the rates of the ripple subtypes in mesio-temporal (amygdale, hippocampal, parahippocampal) and neocortical (frontal, parietal, temporal occipital) regions. The rate per minute of the different ripple subtypes for each channel (all Ripples, SpindleR, SpikeR and Non-SpindleR was calculated. Additionally, the rates in SOZ vs. Non-SOZ channels of each subtype were calculated.

### Correlation With Surgical Outcome

First, we performed a Wilcoxon rank sum test to compare rates of different event types in different brain regions and patient outcomes as listed below:

- mesio-temporal vs. neocortical channels
- SOZ vs. Non-SOZ-channels
- patients with a good post-surgical (Engel I) vs. a bad post-surgical outcome (II–IV).

Significance level was set at α = 0.05.

To evaluate whether the proportion of removed events correlated with the surgical seizure outcome several ratios were calculated between removed and non-removed areas:

1. Ratios between rates of each ripple subtype (ev) in surgically removed channels (RemCh) vs. non-removed channels (Non-RemCh) were calculated for each ripple-subtype (all Ripples, SpindleR, SpikeR, Non-SpindleR), separately.

$$\begin{array}{l} Ratio\;Rate\left(ev\right)=\frac{\sum_{RemCh}Rate\left(ev\right)-\;\sum_{NonRemCh}Rate\left(ev\right)}{\sum_{\left[RemCh,NonRemCh\right]}Rate\left(ev\right)} \end{array}$$

Following the methods of Jacobs et al. (12) a value close to +1 states that the majority of ripples has been removed, and therefore the patient should have a good postsurgical outcome. A value close to −1 states that the majority of ripples remained unchanged, so the postsurgical outcome should be poor. A value around zero indicates that the amount of removed ripples equates approximately the amount of non-removed ripples.

2. Patient-specific thresholds (high-rate ratios) according to the upper-fence-method of Akiyama et al. (40) were calculated to focus on areas with high HFO activity. The-upper-fence-method enabled us to identify channels with high rates of HFO. Ratios for these high-rate channels were calculated in the same way as the ratios for all channels.
3. We calculated if the removal of all SOZ channels (#ChannSOZRem) would result in a better postsurgical outcome than their non-removal (#ChannSOZNonRem).

$$\begin{array}{l} RatioSOZ=\frac{\#ChannSOZRem-\#ChannSOZNonRem}{\#ChannSOZRem+\#ChannSOZNonRem} \end{array}$$

This ratio increases as the proportion of removed to non-removed channels increases. A value close to +1 indicates, that the majority of ripples lay within the SOZ, so after resection patients with a high SOZ-Ratio should have a good postsurgical outcome, if the SOZ and the HFO-generating tissue overlapped. A value close to −1 states that the majority of ripples lay outside the SOZ, these patients should have a poor postsurgical outcome.

Spearman correlations were performed for all described ratios and the post-surgical outcome (Engel I-IV). The significance level of all analyses was set at α = 0.05.

## Results

Thirty one patients met the study inclusion criteria (see Table 1 for clinical information). All patients showed ripples, spikes and sleep spindles in the automatic detections.

**Table 1:** Summary of clinical and demographic data.

**Pat.Nr**.	**Age at OP**	**Gender**	**Type of Seizure**	**MRI**	**Type of surgery**
1	49	M	FAS	no lesion	R sAHC
2	23	F	FAS, FIAS, FBTCS	R HS	R T-pole resection, AHC
3	34	F	FAS, FIAS, FBTCS	NF1, ganglioglioma WHO°I	L F pall lesionectomy
4	44	F	FAS, FIAS, FBTCS	Bil T P MEC	L T-pole resection
5	46	M	FIAS, FBTCS	FCC	R Ant T-pole resection, AHC
6	39	M	FAS, FIAS, FBTCS	R FCD tmp, L T NC lesion	R F lesionectomy
7	60	F	FAS, FIAS, FBTCS	Bil T P MEC, L T P lesion	L T-pole resection, sphen Enceph resection
8	49	M	FAS, FIAS, FBTCS	R O bas FCD	R O T resection
9	51	F	FAS, FIAS, FBTCS	R HS, T A FCD	R Ant T-pole resection, AHC
10	40	F	FAS, FIAS, FBTCS	R HS	R T-pole resection, AHC
11	18	F	FAS, FIAS	L T FCD, HS	L T-pole part resection, AHC
12	25	M	FAS, FIAS, FBTCS	L T M lesion, possible FCD	L lesionectomy, HC
13	12	M	FAS, FIAS	R T possible FCD	R T-pole resection, AHC
14	56	F	FAS, FIAS	R A GC lesion, possible FCD	R Ant lesionectomy GC
15	12	M	FAS, FIAS	tuber sclerosis, NCN	R pall T-pole resection, AHC
16	34	F	FAS, FIAS, FBTCS	R sphen MEC	R T-pole resection, AHC
17	17	M	FAS, FIAS, FBTCS	R T Pol AC, F B lesions	R F resection, T-pole
18	24	F	FAS, FIAS, FBTCS	no lesion	L sAHC
19	52	F	FAS, FIAS, FBTCS	possible postembolic lesions infratent	L T-pole part resection, AHC
20	29	M	FAS, FIAS, FBTCS	Bil T-pol sphen MEC, L possible FCD	L T-pole resection
21	31	F	FAS, FIAS	Bil HS	R T-pole resection, AHC
22	55	M	FAS, FIAS, FBTCS	L T-pol possible FCD	L T-pole part resection
23	27	M	FAS, FBTCS	R possible TAE	R GTS resection
24	55	M	FIAS, FBTCS	L T P MEC, thal infarct 2008	L T-pole resection
25	22	M	FAS	R HS	R sAHC
26	28	F	FAS, FIAS	L FCC, Bil white matter lesions	L T-pole resection, AHC
27	33	F	FAS, FIAS, FBTCS	no lesion	R T-pole part resection
28	53	M	FAS, FIAS, FBTCS	R T-pol possible FCD	R T-pole part resection, AHC
29	38	M	FAS, FIAS	Bil HS	L pall sAHC
30	22	F	FAS, FIAS, FBTCS	PCA WHO°I	R T resection, AHC
31	13	M	FAS, FIAS	no lesion	R T-pole resection, AHC

*AC, arachnoidal cyst; AHC, amygdalohippocampectomy; Ant, anterior; B, basal; Bil, bilateral; Enceph, encephalon; F, frontal; FAS, focal aware seizure; FBTCS, focal to bilateral tonic-clonic seizure; FCC, Fissura choroidea cyst; FCD, focal cortical dysplasia; FIAS, focal impaired awareness seizure; GC, gyrus cinguli; GTS, gyrus temporalis superior; HS, hippocampal sclerosis; infratent, infratentorial; L, left; M, mesial; MEC, meningoencephaloceles; NF1, neurofibromatosis 1; NC, Neocortex; NCN, nevus cell nevi; O, occipital; PCA, pylocytic astrocytoma; pol, polar; R, right; sAHC, selective AHC; sphen, sphenoidal; T, temporal; TAE, teleangieectasia; thal, thalamus; tmp, temporomesiopolar*.

In total 2,291 iEEG channels were analyzed, 187 of these were located in mesio-temporal structures, 2,104 in the neocortex. Overall, 767,763 ripples were detected. Of these 82,717 (10.77%) were SpindleR (Spindle-coincident-ripple), 143,416 (18.68%) SpikeR (Spike Ripples), 572,953 (74.63%) Non-SpindleR (ripples outside spindles) and 511,743 (66.65%) Non-cR (ripples not coincident with spikes or spindles). Ripples coincident with spindles and spikes (29,887; 3.89%) were excluded from the analysis as it was unclear whether to classify them as physiological or epileptic.

In total, 457,995 (59.65%) ripples were found in the temporal neocortex (TNC), followed by the frontal neocortex (FNC: 128688 ripples; 16.76%), the occipital neocortex (ONC: 50,914 ripples; 6.63%), the hippocampus (HC: 46,397 ripples; 6.04%), the parietal neocortex (PNC: 38,642 ripples; 5.03%), the amygdala (A: 31,264 ripples; 4.07%) and the parahippocampal structures (PHC: 13,863 ripples; 1.81%).

### Ripple Distribution Across Brain Regions

**Figure 3:**
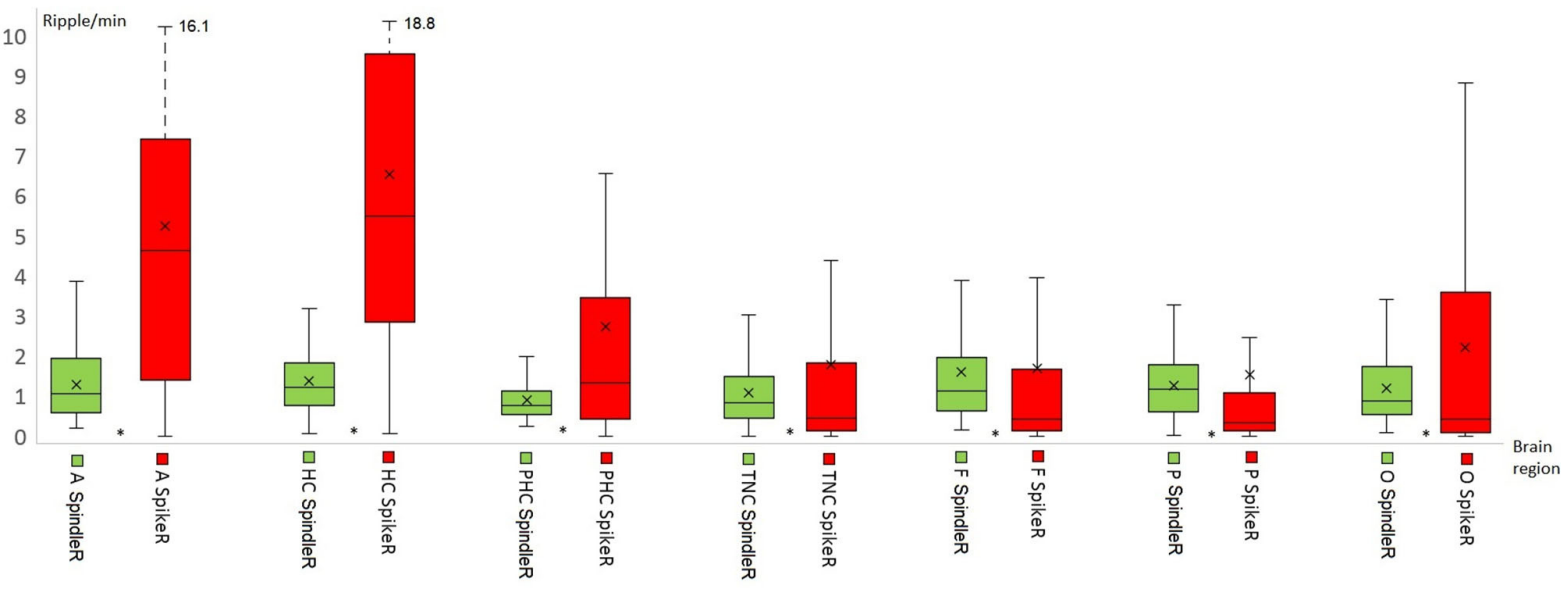
Distribution of Spindle-Ripple (SpindleR) and Spike-Ripple (SpikeR) in different brain areas. SpindleR and SpikeR were more frequent in mesio-temporal structures than in neocortical structures. Mesio-temporal lobe: A, amygdala; HC, hippocampus; PHC, parahippocampal; Neocortex: TNC, temporal neocortex; F, frontal neocortex; P, parietal neocortex; O, occipital neocortex.

**Figure 4:**
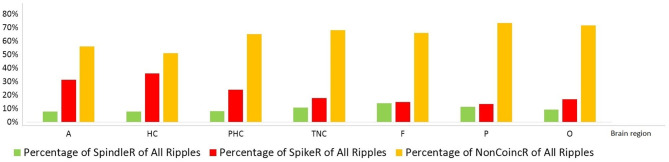
Percentage distribution of the three different ripple subtypes in mesio-temporal and neocortical channels. The amount of SpikeR was higher in mesio-temporal structures compared to the amount of SpindleR, whereas the amount of SpikeR and SpindleR was more alike in the neocortex. Mesio-temporal lobe: A, amygdala; HC, hippocampus; PHC, parahippocampal; Neocortex: TNC, temporal neocortex; F, frontal neocortex; P, parietal neocortex; O, occipital neocortex.

Table 2 shows all rates of the ripple subtypes in different brain regions (see Table 2 in for detailed information), Figure 3 additionally illustrates the distribution of SpindleR and SpikeR. Figure 4 shows the percentage of SpindleR, SpikeR, and Non-CoincidentRipples of the sum of ripples in the specific brain regions, respectively.

**Table 2:** Average ripple rate/minute + SD (standard deviation) for All Ripple, SpikeR, SpindleR, and Non-SpindleR.

**Ø Ripple Rate/min**	**All Ripple (R)**	**SpikeR**	**SpindleR**	**R outside Spindles**
A	4.14 ± 2.67	1.95 ± 3.44	1.20 ± 1.00	9.26 ± 7.49
HC	4.27 ± 2.76	2.07 ± 3.53	1.19 ± 1.00	9.53 ± 7.71
PHC	4.11 ± 2.73	1.97 ± 3.42	1.12 ± 0.85	9.25 ± 7.48
TNC	4.28 ± 2.78	2.09 ± 3.59	1.20 ± 1.00	9.54 ± 7.75
FNC	4.15 ± 2.69	1.96 ± 3.43	1.19 ± 1.00	9.31 ± 7.51
PNC	3.83 ± 2.60	1.83 ± 3.24	1.09 ± 0.82	8.57 ± 7.13
ONC	4,32 ± 2,81	2.13 ± 3.63	1.21 ± 1.01	9.63 ± 7.83

*A, Amygdala; HC, Hippocampus; PHC, Parahippocampus; TNC, Temporal Neocortex; FNC, Frontal Neocortex; PNC, Parietal Neocortex; ONC, Occipital Neocortex*.

Notably none of the ripple subtypes was exclusive to one brain region. Ripples associated with spindles were visible over all brain regions and not exclusively observed in the mesio-temporal structures.

**Figure 5:**
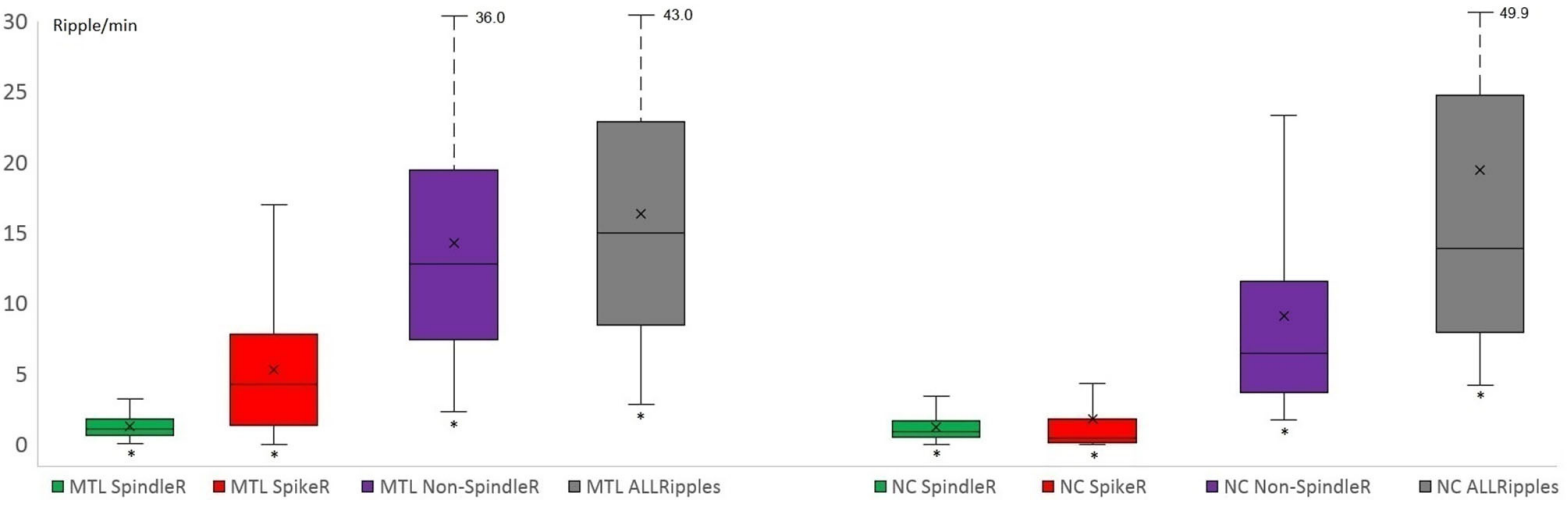
Rates of four different ripple subtypes in mesio-temporal and neocortical channels. Mesio-temporal (MTL) channels showed significantly higher rates of all four ripple subtypes than neocortical (NC) channels. The share of likely pathological ripples [Spike-ripple (SpikeR) and ripples outside spindles (Non-SpindleR)] was significantly higher in the mesio-temporal contacts. SpindleR, Spindle-Ripples.

**Figure 6:**
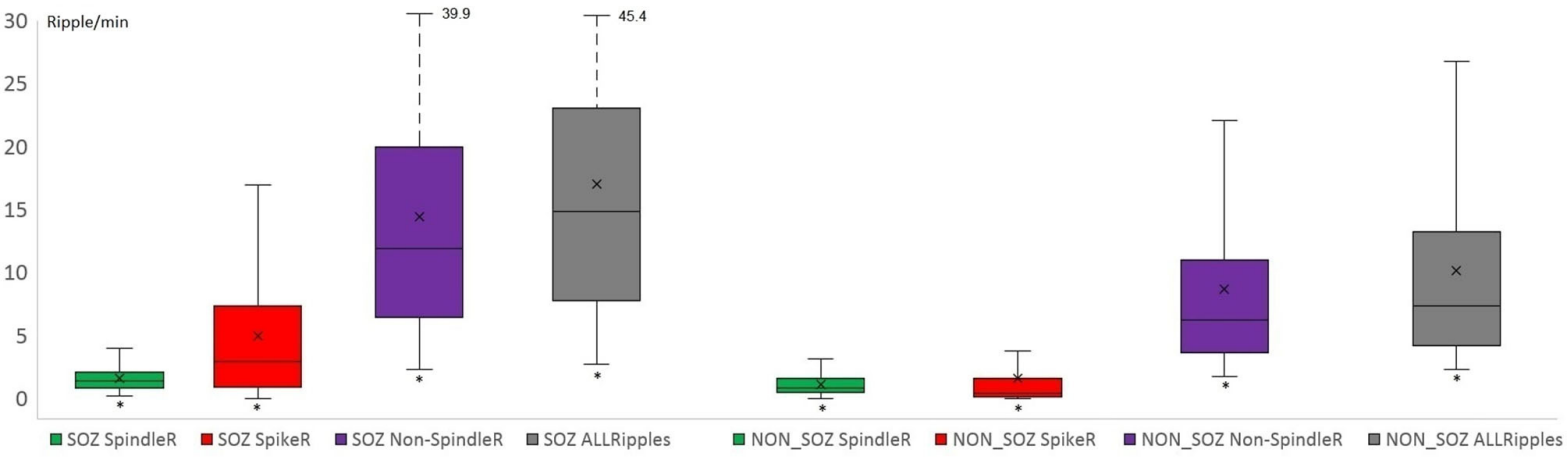
Rates of different ripple subtypes in SOZ and Non-SOZ channels. All Ripple subtype rates were significantly higher in SOZ (=seizure onset zone) channels than in Non-SOZ channels. (SpindleR, Spindle-ripples; SpikeR, spike-ripples; Non-SpindleR, ripples outside spindles).

Mesio-temporal channels showed significantly higher rates in all four ripple subtypes than neocortical channels according to the Wilcoxon rank-sum tests (*p* = 0.015 in SpindleR, *p* < 0.001 in all Ripples, Non-SpindleR and SpikeR) (see Figure 5). All four ripple subtypes showed higher rates in the SOZ channels than in Non-SOZ-channels (*p* = 0.039 in SpindleR, *p* < 0.001 in all Ripples, Non-SpindleR, and SpikeR (see Figure 6).

**Figure 7:**
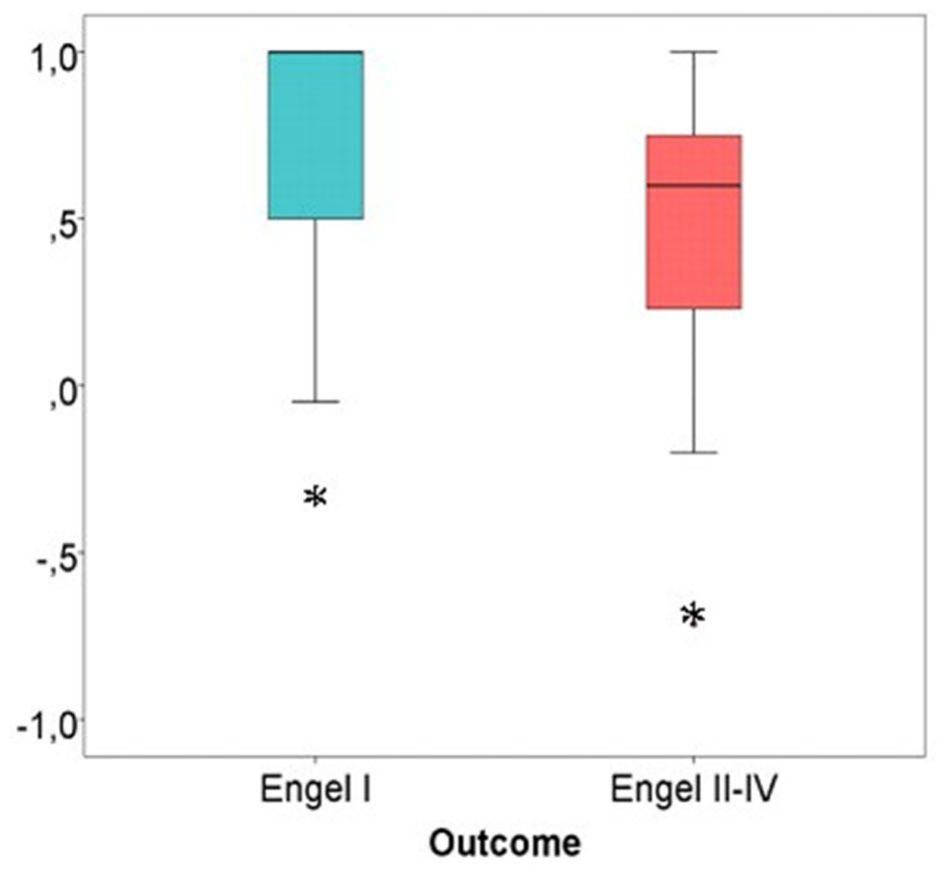
SOZ channel ratio in good vs. bad post-surgical outcome. Patients with the majority of SOZ-channels successfully removed showed a significantly better outcome in comparison to patients with SOZ-channels still in place after resection. Significant difference to the correspondent boxplot (same colour).

The average SOZ ratio over the entire cohort was 0.60 ± 0.43. Patients, in which SOZ channels were removed, showed a significantly better outcome than patients with remaining SOZ channels (Wilcoxon rank sum test: *p* = 0.024) (see Figure 7).

The Spearman correlation concerning the SOZ channel ratio showed a significant correlation between removal of SOZ channels and good outcome: r_s_ = 0.350, *p* = 0.030.

### Correlation Between Surgical Outcome and Removal of HFO Subtypes

The following average rate ratios were obtained for the different ripple subtypes over the entire cohort: All ripples: −0.29 ± 0.33; Non-SpindleR: −0.29 ± 0.33; SpikeR: −0.07 ± 0.46.

**Figure 8:**
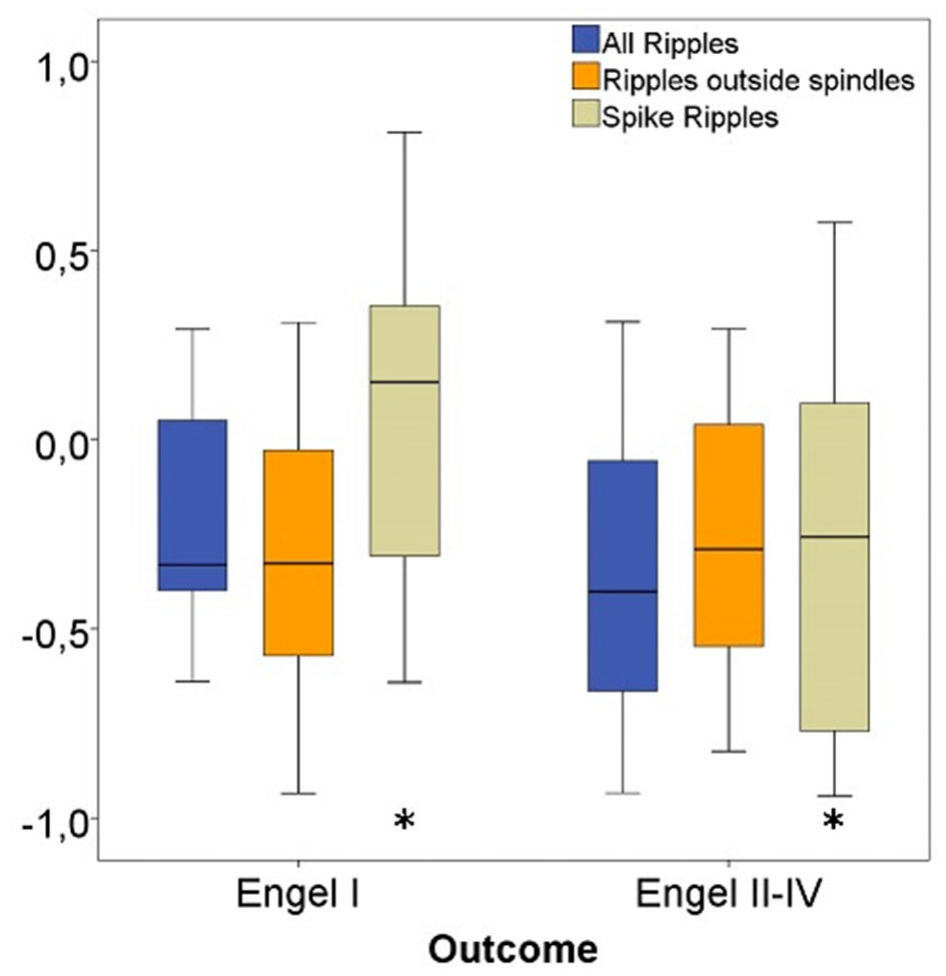
Rate ratios of three ripple subgroups with good vs. bad outcome. The ratio of removed Spike-Ripples were significantly higher in patients with a good (Engel I) compared to patients with a bad postsurgical outcome (Engel II-IV). No significant differences in ratios for Ripples outside spindles and All Ripples between both patient groups were seen. Significant difference to the correspondent boxplot (same colour).

Considering all channels, significantly higher ratios for spike-ripple removal were seen in patients with seizure free vs. poor outcome (*p* = 0.04). No significant differences were seen for the other ripple subtypes (see Figure 8).

**Figure 9:**
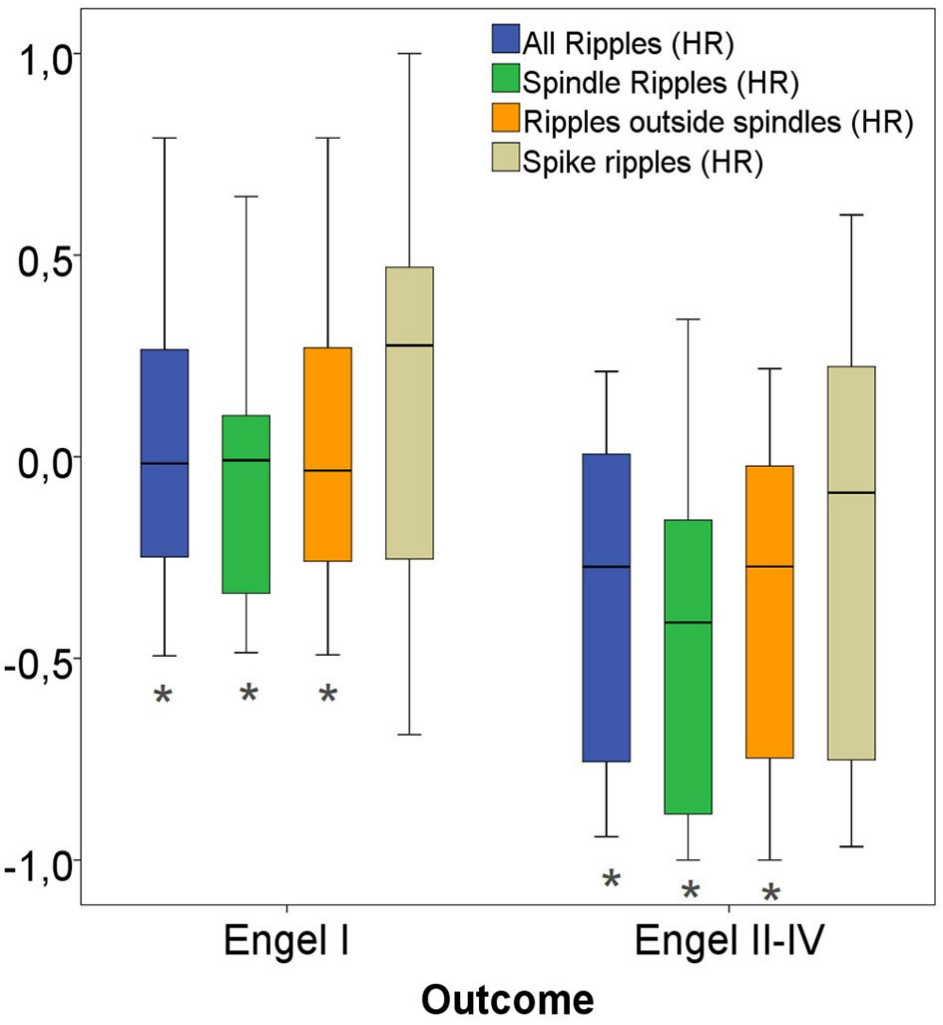
High-rate (HR) ratios of four ripple subgroups with good vs. bad outcome. Significant difference to the correspondent boxplot (same colour).

Considering only channels with high rates of HFO as determined by the upper fence method, a significantly higher proportion of ripples were removed in seizure free patients compared to those with poor outcome. This significant difference was comparable for all ripples (*p* = 0.02), Non-SpindleR (*p* = 0.03) and SpikeR (*p* = 0.04) (see Figure 9).

The Spearman correlations between the removal of the different ripple events and a good post-surgical outcome did not show significant correlations when all channels were analyzed: All ripples (r_s_ = Spearman's rank correlation coefficient = 0.22, *p* = 0.24), Non-SpindleR (r_s_ = 0.16, *p* = 0.39), Spike-ripples (r_s_ = 0.33, *p* = 0.07), ripples not coincident with other events (r_s_ = 0.17, *p* = 0.36), and SpindleR (r_s_ = 0.21, *p* = 0.25).

When only considering high rate channels, the Spearman correlations showed significant correlations between removal of ripple events in high rate ripple channels and seizure free outcome. This was strongest for all ripples (r_s_ = 0.43, *p* = 0.02) and Non-SpindleR (r_s_ = 0.40, *p* = 0.03), but borderline significant for SpikeR (r_s_ = 0.35, *p* = 0.05).

## Discussion

The present study demonstrates that ripples are associated with sleep spindles not only in the mesio-temporal regions but across the brain. Overall, this ripple subtype is rather infrequent and probably only represents a small subpopulation of physiological ripples. As previously described, we could show a correlation between the removal of ripple generating tissue and seizure free outcome. Without restricting the data to areas with frequent ripples, this analysis was only significant for ripples associated with spikes as has been suggested by Roehri et al. (16). Thresholding the data for areas with high ripple rates was highly effective in our population in increasing the correlation between outcome and ripple removal, as has been described previously (40). This correlation was independent of whether we looked at the mixed event group or subpopulations of ripples. Therefore, the separation of spindle associated ripples did not lead to the hypothesized improvement of outcome correlation.

### Occurrence of SpindleR in Different Brain Regions

The first goal of this study was to examine whether spindle associated ripples could be found outside of mesio-temporal structures, as SpindleR have thus far only been reported from mesio-temporal sites (25, 30, 31, 41). Our results suggest that SpindleR can be found across all brain regions. In a second step the anatomic distribution of SpindleR, Non-SpindleR, and SpikeR was assessed.

While it is well-known that the correlation between sleep spindles and ripples in MTL structures is part of the process that allows information transfer from mesio-temporal to neocortical structures (30–32), it remains unclear whether there is a functional spindle-ripple-coupling in neocortical areas. Possibly, neocortical SpindleR fulfill a similar task of information-transfer over wide distances in the brain. However, there is evidence that slow waves, sleep spindles and ripples are functionally connected (42–44). Ellenrieder et al. found a connection of slow waves with mesio-temporal ripples but also with neocortical ripples outside the SOZ (45). Another study showed that neocortical physiological HFO tend to occur with 0.5–1 Hz slow waves, whereas epileptic HFO tend to occur with another type of slow-waves with frequencies between 3 and 4 Hz (22). According to these results it is likely that physiological neocortical ripples may also occur during sleep spindles.

Overall, the proportion of ripples associated with sleep spindles is low. This is the case for contacts inside and outside the SOZ, as well as for contacts with and without epileptic spikes. It is therefore very likely that SpindleR only represent a subtype of physiological ripples expressed in the brain. At this point no study has investigated a correlation between function and SpindleR. It remains therefore an open task to correlate specific functions like memory performance with the proportion of SpindleR expressed over a certain brain region. It will also have to be assessed whether SpindleR are somehow linked to other physiological ripples such as those coupled with slow waves. In the present study a very small percentage of ripples co-occurred with spikes and sleep spindles at the same time. This phenomenon is hard to explain but might be an indicator that physiological ripples occur in epileptic regions and might be visible at the same time as epileptic spikes. This is in line with the observation that SpindleR clearly occur over SOZ areas again suggesting that regions generating physiological and epileptic activity have substantial overlap. This has been suggested by other studies (20), which could not show that high rates of epileptic spikes and HFO necessarily correlate with poor cognitive function.

### Ripple Subtypes in the SOZ

The results of this study showed that all ripple types are most frequent in mesio-temporal regions as described before (6, 45). Moreover, our results are similar to many previous studies in regard to ripple rates being significantly higher inside than outside the SOZ (7, 13, 46–50). As expected, SpikeR and Non-SpindleR showed significantly higher rates in the SOZ. Consistent with recent findings, SpikeR were especially more frequent in SOZ channels than in channels outside the SOZ (51). A previous study from our group suggested that physiological ripples occur and can carry function in SOZ areas (52). It might explain why in this study SpindleR were seen slightly more frequently in the SOZ. This underlines the fact that function can take place in brain areas capable of generating seizures. Moreover, it demonstrates the complexity of separating physiological from epileptic HFO. A pure separation by looking at healthy vs. epileptic brain tissue might fall short of describing the actual coexistence of both event types within the same brain region.

### Correlation Between Surgical Removal of Ripple Generating Areas and the Postoperative Seizure Outcome

It was one important goal of this study to see whether the identification of SpindleR as one group of physiological ripples would improve specificity of HFO as a biomarker for epileptic tissue and therefore improve the prediction of postsurgical seizure outcome. This hypothesis was based on several studies suggesting sleep spindle associated ripples being most probable physiological HFO models (25, 30, 31, 34, 41). The results in this study fail to show clear improvement of outcome correlation when only analyzing those HFO that are not linked to sleep spindles.

As expected, the correlation between HFO removal and surgical outcome was strongest when focusing on spike associated ripples (16, 51). Moreover, results improved when applying a thresholding technique that allows only considering areas with high rates of HFO (20, 40). The categorization of SpindleR therefore does not allow to sufficiently separate physiological and epileptic ripples in all those events that are not coupled to an epileptic spike. The most likely explanation for this observation is that various types of physiological HFO exist and that their characteristics and coupling to other physiological rhythms largely varies as does their function and location.

To actually improve the outcome correlation, it might therefore be essential to combine several techniques to classify ripples as physiological or epileptic. Previous studies suggested that the timing in which ripples are coupled to slow waves is one way to identify physiological ripples (45). Another way might be to analyze several sleep stages as only epileptic ripples are suppressed during phasic REM sleep. Liu and coworkers additionally suggest that epileptic ripples have more stereotypic characteristics than physiological ones (53). The technique presented in this study can identify ripples associated with spikes and sleep spindles in a fully automated way. If these analyses can be combined with other techniques, separation of more physiological ripples might be possible. Independent of this, further research will have to aim at providing a better understanding of influences such as brain region, structural brain abnormalities and epilepsy duration on the occurrence and shape of physiological HFO. The virtual brain atlas project initiated by Frauscher and co-workers is one step in this direction (19).

### Methodological Considerations

There are several limitations of this study, which might have contributed to the above-discussed findings.

First, we only included patients which had electrodes implanted in the mesio-temporal structures as it was unclear at the beginning of this project whether spindle-ripple-association existed outside the mesio-temporal region. This was not limited to patients with temporal lobe SOZ but results in more patients with temporal lobe epilepsy than others. The predominance of contacts in the mesio-temporal structures might have increased the overall number of ripples detected in this study as HFO in general have highest rates in these structures. It is however unlikely that this selection influenced the correlation analysis between ripple removal and outcome, as predominance of ripples in SOZ and surgical areas was visible independent of the location of the SOZ and resection. Additionally, like many previous studies (14) this study had a retrospective design and did not aim to predict surgical outcome prospectively. This design does not provide strong information for translation into clinical use and if a future method of ripple classification should be more successful it would be mandatory to test this method prospectively.

Our study shows that distinct ripple subtypes can be separated by analyzing co-occurrence with spikes and spindles. The analysis was focused on analyzing subtypes of events in each channel, separately analyzing interactions between neighboring or distant channels. At this point it remains unclear whether these subtypes also show distinct network characteristics. While HFO were considered very focal events in the past, most recent research suggests that they might show propagation similarly to the well-described propagation of epileptic spikes. Recent studies have differentiated ripple-subtypes according to their role as “onset-ripples” and “spread-ripples,” suggesting that removing ripples that initiated the propagation (onset-ripples) were associated with a good outcome, whereas removing areas where ripple spread were not (54, 55). Network characteristics and propagation phenomenon of HFO have also been discussed as a result of studies using intraoperative EEG prior and after surgery to analyze HFO. These studies suggest that HFO have network interactions. More specifically HFO visible in the postsurgical EEG might be different in locations from those in the pre-surgical EEG and more relevant for the surgical outcome prediction (50). At this point it is unclear whether HFO networks and propagation is limited to specific anatomical structures and whether network characteristic of HFO are distinct for epileptic and physiological events. Using spindle-ripple analysis could shed future light into this question.

In the present study we focussed on the analysis of scalp sleep spindles using an automated detector that has been modified to application in patients with epilepsy (38, 39). This is important as automated detection tools developed in healthy subjects might not work reliably to detect spindles in scalp EEG that are recorded simultaneous to intracranial EEG in patients with chronic epilepsy. It is well-known that epilepsy influences sleep phenomena and can alter sleep spindle characteristics (56). Epilepsy might reduce the occurrence of sleep spindles. This has been described to be most prominent in times of frequent seizures (57), generalized epilepsies (56), and in those patients with large cortical malformations (58). It can therefore not be excluded that the analyzed patients had reduced sleep spindle occurrence compared to healthy subjects. Most patients however had very focal or no structural abnormalities and we selected EEG periods with the longest time interval away from seizures that could be selected. The fact that we could find sleep spindles and SpindleR in all our patients therefore suggests that SpindleR analysis is possible in patients with chronic refractory focal epilepsy.

## Conclusion and Outlook

The observation that SpindleR occur in brain regions beyond the mesio-temporal areas will be relevant when it comes to understanding functional importance of ripple oscillations as well as using HFO as biomarkers in patients with epilepsy. Extending the questions of this study, spindle-ripple analysis might be useful to identify functionally active brain regions during the pre-surgical diagnostics. A possible correlation of mesio-temporal HFO and memory performance has been examined before (52). An approach for future studies might therefore be the assessment of various cognitive functions and the occurrence of SpindleR.

It has been shown that HFO analysis is not limited to intracranial EEG and that HFO can also be seen in scalp recordings. The identified scalp HFO have a clear intracranial correlate as could be shown in simultaneous scalp-intracranial (59, 60) and EEG-MEG recordings (18). Data suggests that both physiological and epileptic HFO can be seen in scalp EEG (61). Papadelis et al. could show that HFO localization was comparable between invasive and non-invasive methods (62). While scalp HFO clearly co-occur with spikes it is unclear whether there is also a possible temporal coupling with sleep spindles. In the present study no scalp HFO analysis was performed as this is difficult to achieve with automated methods. Moreover, our recordings were too long for visual scalp HFO analysis. Future studies however could focus on scalp HFO spindle correlation and on better understanding which intracranial HFO are visible on the scalp.

Overall, SpindleR are most likely one type of physiological ripple activity generated by the brain. As has been hypothesized, spindle-ripple coupling most likely serves information transfer between brain lobes. Evidence in this study suggests that the value of SpindleR alone to identify physiological ripples on pre-surgical diagnostics is limited. A combination of different methodological approaches including the identification of SpindleR to differentiate between epileptic and physiological HFO is therefore more promising.

## Data Availability Statement

The raw data supporting the conclusions of this article will be made available by the authors, without undue reservation.

## Ethics Statement

The studies involving human participants were reviewed and approved by Ethik-Kommission der Albert-Ludwigs-Universität Freiburg. Written informed consent to participate in this study was provided by the participants' legal guardian/next of kin. Written informed consent was obtained from the individual(s), and minor(s)' legal guardian/next of kin, for the publication of any potentially identifiable images or data included in this article.

## Author Contributions

JJ and JB have contributed to the conception and design of the study. CS, JB, and DL-P have contributed to the acquisition and analysis of data. JB, CS, and JJ have drafted significant portions of the manuscript and figures. AS-B provided all clinical data and EEG data. PR performed the epilepsy surgeries. All authors contributed to the article and approved the submitted version.

## Conflict of Interest

The authors declare that the research was conducted in the absence of any commercial or financial relationships that could be construed as a potential conflict of interest.

## References

[1] Kwan P Brodie MJ Early identification of refractory epilepsy. N Engl J Med. (2000) 342:314–9. 10.1056/NEJM200002033420503 10660394

[2] Schmeiser B Wagner K Schulze-Bonhage A Mader I Wendling A-S Steinhoff BJ. Surgical treatment of mesiotemporal lobe epilepsy: which approach is favorable? Neurosurgery. (2017) 81:992–1004. 10.1093/neuros/nyx138 28582572

[3] Rosenow F Lüders H Presurgical evaluation of epilepsy. Brain. (2001) 124:1683–700. 10.1093/brain/124.9.1683 11522572

[4] Blount JP Cormier J Kim H Kankirawatana P Riley KO Knowlton RC Advances in intracranial monitoring. Neurosurg Focus. (2008) 25:E18 10.3171/FOC/2008/25/9/E18 18759619

[5] Jacobs J LeVan P Châtillon C-É Olivier A Dubeau F Gotman J. High frequency oscillations in intracranial EEGs mark epileptogenicity rather than lesion type. Brain. (2009) 132:1022–37. 10.1093/brain/awn351 19297507 PMC3792079

[6] Jacobs J LeVan P Chander R Hall J Dubeau F Gotman J. Interictal high-frequency oscillations (80–500 Hz) are an indicator of seizure onset areas independent of spikes in the human epileptic brain. Epilepsia. (2008) 49:1893–907. 10.1111/j.1528-1167.2008.01656.x 18479382 PMC3792077

[7] Bragin A Engel J Staba RJ High-frequency oscillations in epileptic brain. Curr Opin Neurol. (2010) 23:151–6. 10.1097/WCO.0b013e3283373ac8 20160649 PMC4063284

[8] Akiyama T McCoy B Go CY Ochi A Elliott IM Akiyama M. Focal resection of fast ripples on extraoperative intracranial EEG improves seizure outcome in pediatric epilepsy. Epilepsia. (2011). 52:1802–11. 10.1111/j.1528-1167.2011.03199.x 21801168

[9] Wu JY Sankar R Lerner JT Matsumoto JH Vinters HV Mathern GW. Removing interictal fast ripples on electrocorticography linked with seizure freedom in children. Neurology. (2010) 75:1686–94. 10.1212/WNL.0b013e3181fc27d0 20926787 PMC3033604

[10] Brázdil M Halámek J Jurák P Daniel P Kuba R Chrastina J. Interictal high-frequency oscillations indicate seizure onset zone in patients with focal cortical dysplasia. Epilepsy Res. (2010). 90:28–32. 10.1016/j.eplepsyres.2010.03.003 20362416

[11] Andrade-Valença L Mari F Jacobs J Zijlmans M Olivier A Gotman J. Interictal high frequency oscillations (HFOs) in patients with focal epilepsy normal MRI. Clin Neurophysiol. (2012) 123:100–5. 10.1016/j.clinph.2011.06.004 21727025 PMC3753287

[12] Jacobs J Zijlmans M Zelmann R Chatillon C-É Hall J Olivier A. High-frequency electroencephalographic oscillations correlate with outcome of epilepsy surgery. Ann Neurol. (2010) 67:209–20. 10.1002/ana.21847 20225281 PMC3769290

[13] Haegelen C Perucca P Châtillon C-E Andrade-Valença L Zelmann R Jacobs J. High-frequency oscillations, extent of surgical resection, and surgical outcome in drug-resistant focal epilepsy. Epilepsia. (2013) 54:848–57. 10.1111/epi.12075 23294353 PMC3712982

[14] Höller Y Kutil R Klaffenböck L Thomschewski A Höller PM Bathke AC. High-frequency oscillations in epilepsy and surgical outcome. A meta-analysis. Front Hum Neurosci. (2015) 9:574. 10.3389/fnhum.2015.00574 26539097 PMC4611152

[15] Jacobs J Kobayashi K Gotman J. High-frequency changes during interictal spikes detected by time-frequency analysis. Clin Neurophysiol. (2011) 122:32–42. 10.1016/j.clinph.2010.05.033 20599418 PMC3774652

[16] Roehri N Pizzo F Lagarde S Lambert I Nica A McGonigal A. High-frequency oscillations are NOT better biomarkers of epileptogenic tissues than spikes. Ann Neurol. (2018) 83:84–97. 10.1002/ana.25124 29244226

[17] Roehri N Bartolomei F. Are high-frequency oscillations better biomarkers of the epileptogenic zone than spikes? Curr Opin Neurol. (2019) 32:213–9. 10.1097/WCO.0000000000000663 30694920

[18] Tamilia E Dirodi M Alhilani M Grant PE Madsen JR Stufflebeam SM. Scalp ripples as prognostic biomarkers of epileptogenicity in pediatric surgery. Ann Clin Transl Neurol. (2020) 7:329–42. 10.1002/acn3.50994 32096612 PMC7086004

[19] Frauscher B von Ellenrieder N Zelmann R Rogers C Nguyen DK Kahane P. High-frequency oscillations in the normal human brain. Ann Neurol. (2018) 84:374–85. 10.1002/ana.25304 30051505

[20] Jacobs J Wu JY Perucca P Zelmann R Mader M Dubeau F. Removing high-frequency oscillations: a prospective multicenter study on seizure outcome. Neurology. (2018) 91:e1040–52. 10.1212/WNL.0000000000006158 30120133 PMC6140372

[21] Nagasawa T Juhász C Rothermel R Hoechstetter K Sood S Asano E. Spontaneous and visually driven high-frequency oscillations in the occipital cortex: intracranial recording in epileptic patients. Hum Brain Mapp. (2012) 33:569–83. 10.1002/hbm.21233 21432945 PMC3220781

[22] Nonoda Y Miyakoshi M Ojeda A Makeig S Juhász C Sood S. Interictal high-frequency oscillations generated by seizure onset and eloquent areas may be differentially coupled with different slow waves. Clin Neurophysiol. (2016) 127:2489–99. 10.1016/j.clinph.2016.03.022 27178869 PMC4867192

[23] Pearce A Wulsin D Blanco JA Krieger A Litt B Stacey WC. Temporal changes of neocortical high-frequency oscillations in epilepsy. J Neurophysiol. (2013) 110:1167–79. 10.1152/jn.01009.2012 23761699 PMC3763087

[24] Blanco JA Stead M Krieger A Stacey W Maus D Marsh E. Data mining neocortical high-frequency oscillations in epilepsy and controls. Brain. (2011) 134:2948–59. 10.1093/brain/awr212 21903727 PMC3187540

[25] Bruder JC Dümpelmann M Piza DL Mader M Schulze-Bonhage A Jacobs-Le Van J. Physiological ripples associated with sleep spindles differ in waveform morphology from epileptic ripples. Int J Neural Syst. (2017) 27:1750011. 10.1142/S0129065717500113 28043201

[26] Frauscher B von Ellenrieder N Ferrari-Marinho T Avoli M Dubeau F Gotman J. Facilitation of epileptic activity during sleep is mediated by high amplitude slow waves. Brain J Neurol. (2015) 138(Pt. 6):1629–41. 10.1093/brain/awv073 25792528 PMC4614129

[27] Buzsáki G. Two-stage model of memory trace formation: a role for “noisy” brain states. Neuroscience. (1989) 31:551–70. 10.1016/0306-4522(89)90423-5 2687720

[28] Buzsáki G The hippocampo-neocortical dialogue. Cereb Cortex. (1996) 6:81–92. 10.1093/cercor/6.2.81 8670641

[29] Diekelmann S Born J The memory function of sleep. Nat Rev Neurosci. (2010) 11:114–26. 10.1038/nrn2762 20046194

[30] Clemens Z Mölle M Eross L Jakus R Rásonyi G Halász P Fine-et altuned coupling between human parahippocampal ripples and sleep spindles. Eur J Neurosci. (2011) 33:511–20. 10.1111/j.1460-9568.2010.07505.x 21138489

[31] Clemens Z Mölle M Eross L Barsi P Halász P Born J. Temporal coupling of parahippocampal ripples, sleep spindles and slow oscillations in humans. Brain J Neurol. (2007) 130(Pt. 11):2868–78. 10.1093/brain/awm146 17615093

[32] Siapas AG Wilson MA. Coordinated interactions between hippocampal ripples and cortical spindles during slow-wave sleep. Neuron. (1998) 21:1123–8. 10.1016/S0896-6273(00)80629-7 9856467

[33] Sirota A Csicsvari J Buhl D Buzsáki G. Communication between neocortex and hippocampus during sleep in rodents. Proc Natl Acad Sci USA. (2003) 100:2065–9. 10.1073/pnas.0437938100 12576550 PMC149959

[34] Piza DL Bruder JC Jacobs J Schulze-Bonhage A Stieglitz T Dumpelmann M. Differentiation of spindle associated hippocampal HFOs based on a correlation analysis. Annu Int Conf IEEE Eng Med Biol Soc. (2016) 2016:5501–4. 10.1109/EMBC.2016.7591972 28269503

[35] Iber C Ancoli-Israel S Chesson AL Quan S The AASM Manual for the Scoring of Sleep and Associated Events: Rules, Terminology and Technical Specifications. Westchest, IL: American Academy of Sleep Medicine (2007).

[36] Staba RJ Wilson CL Bragin A Jhung D Fried I Engel J. High-frequency oscillations recorded in human medial temporal lobe during sleep. Ann Neurol. (2004) 56:108–15. 10.1002/ana.20164 15236407

[37] De Gennaro L Ferrara M Sleep spindles: an overview. Sleep Med Rev. (2003) 7:423–40. 10.1053/smrv.2002.0252 14573378

[38] Lachner-Piza D Jacobs J Bruder JC Schulze-Bonhage A Stieglitz T Dümpelmann M. Automatic detection of high-frequency-oscillations and their sub-groups co-occurring with interictal-epileptic-spikes. J Neural Eng. (2020) 17:016030. 10.1088/1741-2552/ab4560 31530748

[39] Lachner-Piza D Epitashvili N Schulze-Bonhage A Stieglitz T Jacobs J Dümpelmann M. A single channel sleep-spindle detector based on multivariate classification of EEG epochs: MUSSDET. J Neurosci Methods. (2018) 297:31–43. 10.1016/j.jneumeth.2017.12.023 29291925

[40] Akiyama T Otsubo H Ochi A Ishiguro T Kadokura G Ramachandrannair R. Focal cortical high-frequency oscillations trigger epileptic spasms: confirmation by digital video subdural EEG. Clin Neurophysiol. (2005) 116:2819–25. 10.1016/j.clinph.2005.08.029 16253550

[41] Staresina BP Bergmann TO Bonnefond M van der Meij R Jensen O Deuker L. Hierarchical nesting of slow oscillations, spindles and ripples in the human hippocampus during sleep. Nat Neurosci. (2015) 18:1679–86. 10.1038/nn.4119 26389842 PMC4625581

[42] Kudrimoti HS Barnes CA McNaughton BL. Reactivation of hippocampal cell assemblies: effects of behavioral state, experience, and EEG dynamics. J Neurosci. (1999) 19:4090–101. 10.1523/JNEUROSCI.19-10-04090.1999 10234037 PMC6782694

[43] Buzsáki G Rhythms of the Brain. Oxford University Press Available online at: https://www.oxfordscholarship.com/view/10.1093/acprof:oso/9780195301069.001.0001/acprof-9780195301069 (accessed April 19, 2020).

[44] Diba K Buzsáki G. Forward and reverse hippocampal place-cell sequences during ripples. Nat Neurosci. (2007) 10:1241–2. 10.1038/nn1961 17828259 PMC2039924

[45] von Ellenrieder N Frauscher B Dubeau F Gotman J. Interaction with slow waves during sleep improves discrimination of physiologic and pathologic high-frequency oscillations (80-500 Hz). Epilepsia. (2016) 57:869–78. 10.1111/epi.13380 27184021

[46] Jacobs J Staba R Asano E Otsubo H Wu JY Zijlmans M. High-frequency oscillations (HFOs) in clinical epilepsy. Prog Neurobiol. (2012) 98:302–15. 10.1016/j.pneurobio.2012.03.001 22480752 PMC3674884

[47] Cho JR Koo DL Joo EY Seo DW Hong S-C Jiruska P. Resection of individually identified high-rate high-frequency oscillations region is associated with favorable outcome in neocortical epilepsy. Epilepsia. (2014) 55:1872–83. 10.1111/epi.12808 25266626

[48] Kerber K Dümpelmann M Schelter B Le Van P Korinthenberg R Schulze-Bonhage A. Differentiation of specific ripple patterns helps to identify epileptogenic areas for surgical procedures. Clin Neurophysiol. (2014) 125:1339–45. 10.1016/j.clinph.2013.11.030 24368032

[49] Okanishi T Akiyama T Tanaka S-I Mayo E Mitsutake A Boelman C. Interictal high frequency oscillations correlating with seizure outcome in patients with widespread epileptic networks in tuberous sclerosis complex. Epilepsia. (2014) 55:1602–10. 10.1111/epi.12761 25196064

[50] van Klink NEC Van't Klooster MA Zelmann R Leijten FSS Ferrier CH Braun KPJ. High frequency oscillations in intra-operative electrocorticography before and after epilepsy surgery. Clin Neurophysiol. (2014) 125:2212–9. 10.1016/j.clinph.2014.03.004 24704141

[51] Wang S Wang IZ Bulacio JC Mosher JC Gonzalez-Martinez J Alexopoulos AV. Ripple classification helps to localize the seizure-onset zone in neocortical epilepsy. Epilepsia. (2013) 54:370–6. 10.1111/j.1528-1167.2012.03721.x 23106394

[52] Jacobs J Banks S Zelmann R Zijlmans M Jones-Gotman M Gotman J. Spontaneous ripples in the hippocampus correlate with epileptogenicity and not memory function in patients with refractory epilepsy. Epilepsy Behav. (2016) 62:258–66. 10.1016/j.yebeh.2016.05.025 27517349

[53] Liu S Gurses C Sha Z Quach MM Sencer A Bebek N. Stereotyped high-frequency oscillations discriminate seizure onset zones and critical functional cortex in focal epilepsy. Brain. (2018) 141:713–30. 10.1093/brain/awx374 29394328 PMC6715109

[54] Tamilia E Park E-H Percivati S Bolton J Taffoni F Peters JM. Surgical resection of ripple onset predicts outcome in pediatric epilepsy. Ann Neurol. (2018) 84:331–46. 10.1002/ana.25295 30022519

[55] Otárula KAG von Ellenrieder N Cuello-Oderiz C Dubeau F Gotman J. High-frequency oscillation networks and surgical outcome in adult focal epilepsy. Ann Neurol. (2019) 85:485–94. 10.1002/ana.25442 30786048

[56] Drake ME Pakalnis A Padamadan H Weate SM Cannon PA Sleep Spindles in epilepsy. Clin Electroencephalogr. (1991) 22:144–9. 10.1177/155005949102200305 1879053

[57] Tezer FI Rémi J Erbil N Noachtar S Saygi S. A reduction of sleep spindles heralds seizures in focal epilepsy. Clin Neurophysiol. (2014) 125:2207–11. 10.1016/j.clinph.2014.03.001 24684944

[58] Selvitelli M Krishnamurthy K Herzog A Schomer D Chang B. Sleep spindle alterations in patients with malformations of cortical development. Brain Dev. (2008) 31:163–8. 10.1016/j.braindev.2008.06.006 18667284 PMC2722507

[59] Kuhnke N Klus C Dümpelmann M Schulze-Bonhage A Jacobs J. Simultaneously recorded intracranial and scalp high frequency oscillations help identify patients with poor postsurgical seizure outcome. Clin Neurophysiol. (2019) 130:128–37. 10.1016/j.clinph.2018.10.016 30529879

[60] Zelmann R Lina JM Schulze-Bonhage A Gotman J Jacobs J. Scalp EEG is not a blur: it can see high frequency oscillations although their generators are small. Brain Topogr. (2014) 27:683–704. 10.1007/s10548-013-0321-y 24141890

[61] Kuhnke N Schwind J Dümpelmann M Mader M Schulze-Bonhage A Jacobs J. High frequency oscillations in the ripple band (80-250 Hz) in scalp EEG: higher density of electrodes allows for better localization of the seizure onset zone. Brain Topogr. (2018) 31:1059–72. 10.1007/s10548-018-0658-3 29980967

[62] Papadelis C Tamilia E Stufflebeam S Grant PE Madsen JR Pearl PL. Interictal high frequency oscillations detected with simultaneous magnetoencephalography and electroencephalography as biomarker of pediatric epilepsy. J Vis Exp JoVE. (2016) 118:54883. 10.3791/54883 28060325 PMC5226354

